# Moving Beyond Self-Report in Characterizing Drug Addiction: Using Drug-Biased Behavior to Predict Treatment Completion and Dropout in Heroin-Primary, Medication-Maintained Opioid Use Disorder

**DOI:** 10.1016/j.bpsgos.2025.100667

**Published:** 2025-12-09

**Authors:** Natalie McClain, Ahmet O. Ceceli, Kathryn Drury, Greg Kronberg, Eric L. Garland, Nelly Alia-Klein, Rita Z. Goldstein

**Affiliations:** a Department of Neuroscience, Icahn School of Medicine at Mount Sinai, New York, New York; b Department of Psychiatry, Icahn School of Medicine at Mount Sinai, New York, New York; c T. Denny Sanford Institute for Empathy and Compassion La Jolla, San Diego, California; d Department of Psychiatry, University of San Diego La Jolla, San Diego, California

**Keywords:** Addiction, Attention, Behavior, Fluency, Opioids, Self-report

## Abstract

**Background:**

Drug addiction is accompanied by enhanced salience attributed to drug over nondrug cues. This bias can be objectively measured and is reliable but underutilized in informing clinical end points, where self-report measures are most commonly used, with limited success.

**Methods:**

We investigated whether behavioral picture choice (laboratory-simulated measure of drug seeking) and verbal fluency (drug and nondrug words generated) revealed drug-biased processing in 59 individuals with opioid use disorder (iOUDs) compared with 29 healthy control (HC) individuals; assessed twice, we also inspected the test-retest reliability of these tools. All iOUDs were heroin primary, abstinent (160.58 ± 188.18 days), and stabilized on medication for OUD at an inpatient treatment facility at baseline. Then, we tested whether, compared with self-report measures, these drug-biased behavioral measures could better predict prospective outcome measures in the iOUDs, i.e., study treatment completion as further validated using dropout from inpatient treatment.

**Results:**

Results revealed that the iOUDs exhibited higher drug choice (*p*s < .036) and drug fluency (*p* = .008) compared with the HC individuals; task performance demonstrated the strong test-retest reliability of these measures. Controlling for cognitive demographics, the self-report drug-use severity and craving measures did not show significant associations with study treatment completion (|β| < 0.47, *p*s > .290), but drug-biased choice did (β = −0.75, *p* = .036; model comparison: Δ*R*^2^ = 0.10, *p* = .027). Importantly, these results were validated using inpatient treatment dropout as the outcome (drug-biased choice: β = 0.81, *p* = .049; model comparison: Δ*R*^2^ = 0.11, *p* = .035).

**Conclusions:**

This study is the first to demonstrate reliable drug-biased choice and fluency in iOUDs. Compared with traditional self-reported drug-use and craving measures, the objective drug-biased cognitive behavioral measure was a significant predictor of treatment-related outcomes.

## Plain Language Summary

In this study, we examined whether behavioral markers of drug bias provide better prediction of treatment outcomes compared with traditional self-report measures in heroin-primary individuals with opioid use disorder (iOUDs). Results revealed that, compared with healthy control individuals, iOUDs exhibited drug-biased choice and fluency behavior; task performance also revealed high test-retest reliability of these measures. Importantly, drug-biased behavior predicted whether iOUDs completed study treatment or dropped out of inpatient care, while self-reported drug-use severity and craving measures did not. These findings suggest that objective behavioral tasks may offer more reliable markers of severity and indicators of future treatment engagement in OUD.

Overdose remains the leading cause of death for people ages 18 to 44 years (1), largely driven by opioids, including heroin and fentanyl co-use (2,3). Premature treatment dropout is a major obstacle in curbing these overdose deaths, with dropout rates averaging 30% across substances and posttreatment relapse rates as high as 91% in opioid addiction (4, 5, 6). Thus, identifying markers of treatment retention is imperative for relapse vulnerability detection and its prevention. A core symptom that perpetuates drug addiction and deters successful recovery is the attribution of excessive value to drug cues at the expense of nondrug cues and reinforcers (7,8). Measures of this bias toward drugs and away from nondrug rewards may provide a reliable, objective index of addiction severity and serve as a robust predictor of clinical outcomes.

Clinical trials that target addiction treatment have relied heavily on self-report measures of drug use and craving to ascertain addiction severity and treatment end points. While useful in constructing a comprehensive addiction profile [e.g., retrospective patterns of use (9)] and predicting drug use (10) and relapse (11), many self-report tools lack strong psychometric validity for treatment-relevant constructs (12). Additionally, self-report measures are susceptible to reporting errors (13, 14, 15) and can be influenced by compromises in insight into illness (16), demand characteristics (17), and social desirability (18). A study comparing self-report with clinic records in drug treatment patients documented misreporting of heroin use (19), potentially due to social stigma (19,20). The accuracy of self-report may also vary by treatment stage, as evidenced by increasing discrepancies with urine toxicology over time in outpatient opioid detoxification (21), limiting its applicability in long-term treatment settings. Clinical addiction research has also traditionally relied on urine toxicology; however, this measure often fails to capture the complex dynamics of drug-use patterns (22) and loses utility in controlled treatment or correctional settings where drug urine results are invariably negative. Therefore, additional approaches are needed to better characterize clinical end points in drug addiction treatment, especially for opioid addiction, which affects the majority of those seeking treatment for drug use (23,24).

While underutilized, there is mounting evidence that objective cognitive behavioral measures enhance disease characterization and outcome prediction in addiction, particularly when tailored toward drug contexts (25). Nondrug neuropsychological and behavioral measures of impulsivity (26,27), decision making (28,29), attention, visual-verbal memory, and visuospatial ability (30) have been shown to predict clinical end points in people with substance use disorders/dependence. Tasks that assess motivated attention toward drug-related stimuli may be particularly valuable in predicting outcomes. Behavioral measures tailored to drug-related contexts (e.g., drug Stroop and visual probe tasks) yield a drug bias (31) that predicts temptations to use (32), relapse (33,34), misuse risk (35), prospective use (36), and treatment engagement (36,37) in people with substance use disorders/dependence. Beyond attention, drug-biased picture choice has predicted recent and prospective (6 months) drug use in cocaine addiction (38, 39, 40) and prospective (8 weeks) misuse severity in opioid-treated pain patients (41). Drug picture choice has also predicted opioid use disorder (OUD) symptom severity in pain patients with prescription OUD (42). Drug-related speech has also predicted longitudinal outcomes (craving, withdrawal, abstinence, drug use), outperforming demographic, neuropsychological, and self-report drug-use measures in predicting 12-month abstinence in cocaine addiction (43). Taken together, cognitive behavioral tasks that target higher-order drug-biased processing offer sensitive and reliable proxies for drug-use severity that, compared with self-report or nondrug measures, may better predict clinical outcomes also in individuals with OUD (iOUDs). However, no previous work has tested whether drug-biased cognitive behavioral measures can outperform self-report in predicting objectively measured treatment-related outcomes in iOUDs, as investigated in the current study.

Here we tested whether select cognitive behavioral markers of drug bias outperform cognitive demographic and traditional self-report measures of addiction severity in informing prospective (4 months) treatment completion and dropout in inpatient, heroin-primary iOUDs stabilized on medication for opioid use disorder (MOUD). Probing attentional bias and conditioned reactivity toward drug-related stimuli, the behavioral markers included 1) picture-viewing choice tasks [demonstrated in cocaine addiction (39,40) and validated across other drugs, including prescription opiate misuse in pain patients, methamphetamines, and nicotine (41,42,44,45)] and 2) a verbal fluency task [demonstrated in cocaine addiction (46,47)] designed to index bias toward drug words. Attendance of a follow-up study visit immediately after a randomized treatment phase served as the primary outcome measure of study treatment completion. To further validate these results, participant dropout from primary inpatient treatment [defined as leaving against clinical advice (48)] served as a secondary outcome measure. We hypothesized that 1) the iOUDs would show higher drug-biased choice and fluency compared with healthy control (HC) individuals and 2) these objective drug bias measures would outperform cognitive demographic, self-report drug use, and craving measures in informing treatment-related outcomes. As an exploratory hypothesis, we anticipated test-retest reliability in these drug-biased behaviors in iOUDs.

## Methods and Materials

### Participants

**Figure 1:**
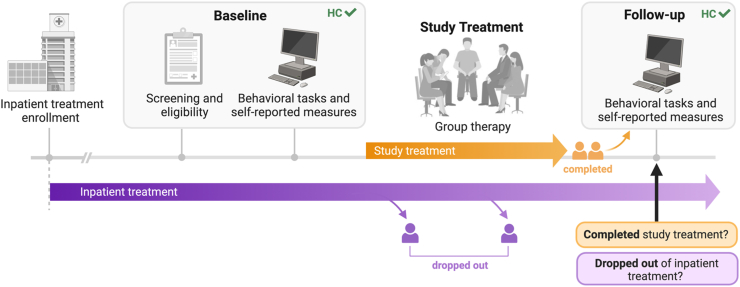
A timeline depicting study events that occurred alongside inpatient treatment in individuals with opioid use disorder (iOUDs). All iOUDs were enrolled in inpatient treatment at baseline, when the behavioral and self-report measures were collected following screening. After baseline, iOUDs partook in the study treatment (group therapy), which concluded with the follow-up visit, when the outcome measures were assessed (study treatment completion and inpatient treatment dropout). The healthy control (HC) check icons indicate the study events that involved HC individuals. Arrows branching off inpatient treatment represent dropout from or completion of inpatient treatment while participating in the study.

Fifty-nine iOUDs and 29 age- and sex-matched HC individuals from the surrounding community participated in this study (see Table 1). At baseline, all iOUDs were enrolled at an inpatient drug treatment program (see the Supplement for details) and met criteria for OUD with heroin as their primary substance. At baseline, all iOUDs were abstinent (160.58 ± 188.18 days) confirmed via urine toxicology, with 4 participants testing positive for nonsynthetic opiates at follow-up. All iOUDs were stabilized on MOUD (methadone: *n* = 52, 112.94 ± 60.75 mg; suboxone: *n* = 7, 13.33 ± 8.64 mg), initiated upon inpatient enrollment (257.29 ± 764.07 days before baseline) and maintained throughout the study (confirmed via urine toxicology) (see Figure 1). See the Supplement for details regarding eligibility criteria, diagnostic interviews, psychiatric diagnoses and comorbidities, and the assessments presented in Table 1.

**Table 1:** Sample Profile at Baseline

	HC, *n* = 29	OUD, *n* = 59	*p* Value
Demographics			
Age, years	40.70 (10.64)	41.39 (9.52)	.564
Sex, female/male/other	10/19/0	14/44/1	.463
Race, Black/White/other	12/13/4	5/45/9	.001∗
Education, years	16.22 (2.94)	12.10 (2.01)	<.001∗
Verbal IQ	108.66 (7.30)	95.54 (10.72)	<.001∗^,^^a^
Nonverbal IQ	11.69 (3.06)	9.76 (3.23)	.005
Handedness, right/left	22/7	46/13	.825
Self-Report Drug-Use Severity			
Regular opioid use, years	–	11.20 (6.94)	–
Heroin use past month, days	–	0.20 (0.94)	–
SDS	–	11.66 (3.35)	–
SOWS	–	3.56 (5.51)	–
Self-Report Craving			
HCQ	–	40.62 (15.01)^b^	–
Picture cue-induced craving	–	1.99 (1.08)^c^	–
Movie scene-induced craving	–	1.16 (1.06)^d^	–
STRAP-R	–	0.54 (1.60)	–
Other Substance Use			
Cigarette smoking status, current/past/never	1/4/24	56/3/0	<.001∗
FTND	1 (0)	3.45 (1.75)	.027
Regular marijuana use, years	0.80 (1.65)	8.43 (9.63)	<.001∗
Regular alcohol use, years	9.60 (12.52)	8.05 (10.20)	.652
Heroin administration, injection/nasal/oral/smoking	–	34/21/1/3	–
Medication for OUD, methadone/suboxone	–	52/7	–
Depression and Anxiety			
Beck Depression Inventory	2.97 (4.47)	14.21 (11.38)^e^	<.001∗
Beck Anxiety Inventory	2.00 (3.82)	10.10 (10.01)^f^	<.001∗

Values are presented as *n* or mean (SD). Wilcoxon rank-sum tests assessed group differences for continuous variables. χ^2^ tests were used for unordered categorical and binary data. Smoking status was excluded from this correction due to the near parallel distribution matching group identity. All measures were collected at the baseline study visits.

∗Significant between-group differences corrected for familywise error (α = 0.05/12 = 0.0042).

FTND, Fagerström Test for Nicotine Dependence; HC, healthy control; HCQ, Heroin Craving Questionnaire; OUD, opioid use disorder; SDS, Severity of Dependence Scale; SOWS, Subjective Opiate Withdrawal Scale; STRAP-R, Sensitivity to Reinforcement of Addictive and Other Primary Rewards.

a Welch’s *t* test compared group differences due to violations of homogeneity of variances.

b One missing HCQ score.

c One missing cue-induced craving score.

d Two missing scene-induced craving scores.

e One missing Beck Depression Inventory score.

f One missing Beck Anxiety Inventory score.

Participants provided informed consent in accordance with Mount Sinai’s Institutional Review Board and were compensated for study procedures. Data were collected from October 21, 2020, to February 1, 2023.

### Drug-Related Cognitive Behavioral Tasks

#### Drug Choice

The choice to view pleasant (e.g., people smiling), unpleasant (e.g., wounds), neutral (e.g., office supplies), and drug (e.g., people using/preparing heroin) pictures was assessed explicitly and probabilistically. In the explicit version, participants chose between 2 side-by-side images (from these 4 categories and blank images) via button presses. A single press expanded the corresponding image for 0.5 seconds, while repeated presses allowed the image to remain expanded for the duration of the trial (5 seconds), such that total responses per category indexed viewing effort (completion time: 350 seconds). In the probabilistic version, participants picked 1 card from 4 flipped-over, pseudorandomly ordered decks, each containing primarily one category (e.g., drug), including 2 pictures from another category (e.g., pleasant) and one from each of the remaining categories. Choosing a card displayed the image full screen for 2 seconds. Selecting the same dominant category 8 times ended the run, after which deck positions were reconfigured [similar to (49)], requiring participants to relocate their preferred deck. These contingencies minimized recognition of deck identity while still allowing participants to establish deck preference (completion time depended on time to establish deck preference) (39). For further details, see Moeller *et al.* (39).

#### Drug Fluency

Participants were instructed to generate as many drug words (names of people, places, or mind states related to obtaining, using, or recovering from drugs) as possible in 1 minute (46,47). The standard semantic fluency task was administered to measure nondrug fluency, where participants were asked to name as many animals and fruits/vegetables as possible for 1 minute per category (completion time: 3 minutes) (50).

These tasks were performed at baseline and follow-up (119 ± 56 days apart), with no group differences in the number of days between sessions (iOUD: 108 ± 36; HC: 136 ± 76; *z* = −1.35, *p* = .178) (Figure 1).

### Treatment Completion and Dropout

Two treatment-related outcome measures were selected, the first of which was study treatment completion. After completing the baseline assessments and supplemental to their inpatient treatment, the iOUDs participated in a clinical trial (NCT04112186) in which they were randomly assigned to an 8-week group therapy intervention [Mindfulness Oriented Recovery Enhancement (*n* = 28) (51) or a support group (*n* = 30); 1 participant discharged prior to randomization; therapy-specific effects will be reported separately]. A posttreatment follow-up visit marked study treatment completion (completed = 1, did not complete = 0) and involved repeating the baseline tasks (see Figure 1) and was scheduled individually to optimize attendance. In the iOUDs, reasons for study treatment noncompletion (*n* = 16) reflected common barriers to treatment adherence (e.g., unresponsiveness to scheduling efforts [*n* = 10], unwillingness to continue [*n* = 4], study noncompliance [*n* = 2]). Consistent with this interpretation, study treatment completers (*n* = 43) were more engaged throughout the study treatment, completing a greater number of therapy sessions (completers: 6 ± 2; noncompleters: 2 ± 2; *z* = −4.68, *p* < .001) and daily assessments (completers: 23 ± 20; noncompleters: 6 ± 9; *W* = −2.91, *p* = .004). Those who were outpatient by study treatment completion were discharged 128.57 ± 134.29 days prior to follow-up. HC participants were studied at similar intervals, and all attended both baseline and follow-up sessions, yielding significant group differences in follow-up attendance rates (χ^2^_1,__*n*__= 88_ = 7.88, *p* = .005).

Inpatient treatment dropout was the second treatment-related outcome. The secondary outcome indexed a more direct treatment-relevant end point. Collected from inpatient treatment records, dropout from inpatient treatment was defined as leaving against clinical advice prior to follow-up (dropped out = 1, did not drop out = 0). Here, 11 iOUDs dropped out of inpatient treatment, on average 57 ± 34 days after baseline. The iOUDs who did not drop out of inpatient treatment (*n* = 48) either completed inpatient treatment (*n* = 9), were administratively discharged (*n* = 1), or were still inpatient (*n* = 38) at follow-up.

Lower inpatient treatment dropout was associated with study treatment completion (χ^2^_1,__*n*__= 59_ = 24.01, *p* < .001); the study treatment completers had a greater proportion of those who did not drop out (42 did not drop out vs. 1 dropped out), while the noncompleters had a greater proportion of those who dropped out of inpatient treatment (6 did not drop out vs. 10 dropped out), further validating study treatment completion as a clinically relevant outcome measure.

### Statistical Analysis

#### Choice Behavior and Fluency

The total response count and total image selections per picture category across all trials for the explicit and probabilistic choice tasks, respectively, yielded category-specific choice. The a priori selected drug > pleasant contrast estimated drug-biased choice (controlling for a nondrug reward). Drug and nondrug fluency scores were calculated by summing correct responses per minute, excluding repetitions and errors (i.e., a sum for the 1-minute drug category and an average across the two 1-minute nondrug categories). The drug > nondrug contrast represented drug-biased fluency.

Three mixed analyses of variance (ANOVAs) were conducted for the behavioral variables at baseline: 1) for explicit choice, a 2 (group: iOUD, HC) × 5 (cue type: drug, pleasant, unpleasant, neutral, blank) ANOVA; 2) for probabilistic choice, a 2 (group: iOUD, HC) × 4 (cue type: drug, pleasant, unpleasant, neutral) ANOVA (2 iOUDs and 1 HC individual missing); and 3) for fluency, a 2 (group: iOUD, HC) × 2 (cue type: drug, nondrug) ANOVA (1 iOUD missing), excluding missing data. For all 3 measures, longitudinal effects were estimated using mixed ANOVAs with an additional session factor (baseline, follow-up); correlations between baseline and follow-up behavior were also conducted (see the Supplement).

Significant interactions (*p* < .05) were followed by paired and independent parametric *t* tests. Nonparametric (Wilcoxon and Welch’s) *t* tests were used when assumptions of normality and homogeneity of variance were violated, respectively. To summarize results across multiple tests, we report the smallest absolute *t* or *z* statistic for significant effects and thresholds for nonsignificant results.

To test their contribution, measures with significant group differences (Table 1) were correlated with the primary behavioral measures; those with significant associations were entered into analyses of covariance (see the Supplement).

#### Dimension Reduction and Hierarchical Logistic Regressions

We used a dimension reduction approach to test whether the latent constructs aligned with our hypothesized categories of predictor variables: 1) cognitive demographics, 2) self-report drug-use severity, 3) self-report craving, and 4) drug-biased behavior. Considering the total number of participants and our dimensions of interest, we aimed to reduce the number of predictors to 4. Thus, we conducted an exploratory factor analysis using the continuous variables [see (52)] from the first 3 categories in Table 1, as well as the primary behavioral measures (drug > pleasant explicit and probabilistic choice and drug > nondrug fluency). For each factor, the highest-loading variable was selected as the representative predictor, yielding one predictor per category. See the Supplement for further details.

Then, we performed two hierarchical logistic regression analyses using study treatment completion and inpatient treatment dropout as outcome measures. Predictors were added stepwise in the order specified above to test their unique contributions, with likelihood ratio tests used for model comparisons.

There were no significant differences in demographic or drug-use variables (see Table 1) between the study treatment completion and inpatient dropout iOUD subgroups (see the Supplement); therefore, no additional measures were controlled for a priori in the primary regression analyses (see the Supplement for post hoc analyses).

All statistical analyses were conducted in R (version 4.4.2) (see the Supplement for functions and software packages).

## Results

### Fluency and Choice Behavior

**Figure 2:**
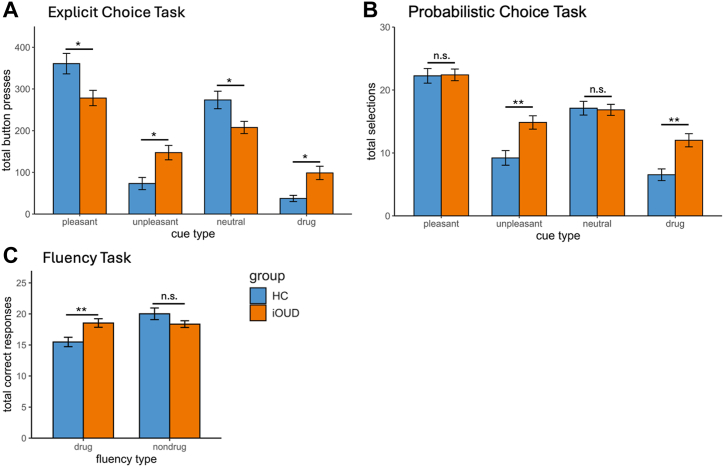
Choice and fluency behavior at baseline. Individuals with opioid use disorder (iOUDs) and healthy control (HC) participants’ **(A)** explicit choice behavior, indicating a bias in iOUDs toward drug (*p* = .036) and unpleasant (*p* = .014) cue types and a bias in HC participants toward pleasant (*p* = .010) and neutral (*p* = .011) cue types; **(B)** probabilistic choice behavior, indicating a bias in iOUD toward drug (*p* = .002) and unpleasant (*p* = .002) cue types, with no group differences in pleasant (*p* = .840) and neutral (*p* = .857) cue types; and **(C)** verbal fluency, indicating drug-biased fluency in iOUDs (*p* = .008), with no group differences in nondrug fluency (*p* = .103). Blank cue results are not displayed. Error bars indicate SEM. Asterisks reflect significant group differences: ∗*p* < .05, ∗∗*p* < .01. n.s., nonsignificant.

Explicit choice results revealed no main effect of group (*F*_1,86_ = 0.72, *p* = .399), a main effect of cue type (pleasant > neutral > blank = unpleasant > drug; *F*_4,344_ = 76.61, *p* < .001), and a group × cue type interaction (*F*_4,344_ = 10.75, *p* < .001). This interaction was driven by more drug and unpleasant image choice (*z* > 2.09, *p* < .037) and less pleasant, neutral, and blank image choice (*t*_86_ > 2.58 or *z* > 2.43, *p* < .015) in the iOUD group compared with the HC group; within groups, drug image choice was lower than choice for all other categories (iOUD: *z* > 3.00, *p* < .003; HC: *z* > 2.07, *p* < .039) (Figure 2A).

Probabilistic choice results revealed a main effect of group (iOUD > HC; *F*_1,83_ = 8.10, *p* = .006) and cue type (pleasant > neutral > unpleasant > drug; *F*_3,249_ = 61.79, *p* < .001) and a group × cue type interaction (*F*_3,249_ = 4.92, *p* = .002). This interaction was driven by more drug and unpleasant (*z* > 3.12, *p* < .002) image choice in iOUDs compared with HC individuals, with no group differences for pleasant or neutral images (*t*_83_ = 0.18 or *z* = 0.20, *p* < .857); within groups, selection of drug images was lower than choice for all other categories (iOUD: *z* > 3.79, *p* < .001; HC: *z* > 3.30, *p* < .001) (Figure 2B).

Fluency results revealed no main effect of group (*F*_1,85_ = 0.61, *p* = .439), a main effect of cue type (nondrug > drug; *F*_1,85_ = 14.24, *p* < .001), and a group × cue type interaction (*F*_1,85_ = 16.70, *p* < .001), revealing more drug word generation by iOUDs compared with HC individuals (*z* = 2.64, *p* = .008), with no difference in nondrug words (*t*_85_ = 1.65, *p* = .103); within groups, the HC individuals generated more nondrug than drug words (*t*_28_ = 5.00, *p* < .001), which was not observed in iOUDs (*z* = 0.01, *p* = .990) (Figure 2C).

Group differences in Table 1 measures did not contribute to the baseline ANOVA results (see the Supplement). There were no session-related effects detected for any of the tasks (*p*s > .113) (see the Supplement), and within-subject correlations revealed associations between baseline and follow-up behavior (*p*s < .010) (see the Supplement).

### Dimension Reduction and Hierarchical Logistic Regressions

The factor analysis for dimension reduction yielded 4 factors where the highest-loading variables were consistent with our hypothesized constructs: education (cognitive demographics; λ = 0.52), regular opioid use (self-report drug-use severity; λ = 0.95), picture cue-induced craving (self-report craving; λ = 0.93), and drug > pleasant explicit choice (drug-biased behavior; λ = 0.99) (Table 2).

**Table 2:** Dimension Reduction Results

	Factor Loadings				*h*^2^	1 − *h*^2^
	1	2	3	4		
Cognitive Demographics						
Age	0.48	−0.05	−0.14	−0.01	0.25	0.75
Education, years	−0.14	0.10	−0.09	0.52^a^	0.31	0.70
Verbal IQ	−0.03	0.16	0.04	0.50	0.28	0.72
Nonverbal IQ	0.07	0.19	0.06	0.45	0.25	0.75
Self-Report Drug-Use Severity						
Regular opioid use, years	0.95^a^	0.14	0.21	0.16	1.00	0.00
SDS	−0.01	0.02	0.08	−0.14	0.03	0.97
SOWS	0.34	−0.10	−0.02	−0.04	0.03	0.87
Self-Report Craving						
HCQ	0.05	0.04	0.34	−0.25	0.19	0.81
Picture cue-induced craving	−0.33	−0.01	0.93^a^	0.12	1.00	0.00
Movie scene-induced craving	−0.04	−0.11	0.54	−0.06	0.30	0.70
STRAP-R	0.42	0.10	−0.11	−0.08	0.20	0.80
Drug-Biased Behavior						
Drug > pleasant explicit choice	−0.02	0.99^a^	−0.03	0.08	1.00	0.00
Drug > pleasant probabilistic choice	0.20	0.52	0.18	−0.32	0.44	0.56
Drug > nondrug fluency	−0.10	0.41	−0.22	0.12	0.24	0.76
Eigenvalue	1.62	1.55	1.45	0.98	–	–
% of Variance	29%	28%	26%	18%	–	–
Cumulative %	29%	57%	82%	100%	–	–

Factor loadings, communality (*h*^2^), uniqueness (1 − *h*^2^), eigenvalues, percentages of variance and cumulative percentages of variance are shown. The extraction method used was maximum likelihood estimation with varimax rotation. This was conducted in 52 individuals with opioid use disorder with complete data for all included measures (past-month heroin use was excluded due to lack of variance).

HCQ, Heroin Craving Questionnaire; SDS, Severity of Dependence Scale; SOWS, Subjective Opiate Withdrawal Scale; STRAP-R, Sensitivity to Reinforcement of Addictive and Other Primary Rewards.

a Highest loadings within each factor.

The hierarchical regression results revealed that the first model with the cognitive demographic measure (education) alone did not significantly predict study treatment completion (*R*^2^ = 0.06, *p* = .097; β = 0.58, *p* = .122). The second model including the self-report drug-use severity measure (regular opioid use) also did not significantly predict treatment completion (*R*^2^ = 0.08, *p* = .191) and did not perform significantly better compared with the cognitive demographics model (Δ*R*^2^ = 0.014, *p* = .457). Similarly, the third model including the self-report craving measure (picture cue-induced craving) was not significant (*R*^2^ = 0.08, *p* = .312) and did not perform better compared with the previous model (Δ*R*^2^ = 0.003, *p* = .611). However, adding the drug-biased behavior measure (drug > pleasant explicit choice) significantly increased the predictive strength of the model in informing treatment completion, increasing the pseudo-*R*^2^ by 10.2% (*p* = .027). In this final model, the drug-biased behavior measure significantly predicted treatment completion such that the higher the baseline drug bias, the lower the likelihood of completion (β = −0.75, *p* = .036), which was not observed for the cognitive demographic, self-report severity, or self-report craving measures (*p*s > .061). In the final model, 18% of the variance in the likelihood of treatment completion was associated with the regressors (*p* = .075) (Table 3). A post hoc regression including only the drug-biased behavior measure, controlling for cognitive demographics, significantly predicted study treatment completion (Akaike information criterion [AIC] = 59.9, pseudo-*R*^2^ = 0.13, *p* = .035); this effect was not observed for the self-report severity and craving measures (*p*s > .191). Without controlling for cognitive demographics, the model with the behavioral measure alone approached significance (AIC = 61.4, pseudo-*R*^2^ = 0.09, *p* = .075).

**Table 3:** Hierarchical Logistic Regression Analysis for Study Treatment Completion

	β	SE	*p*	Model Summary			Model Comparison		
				AIC	*R*^2^	*p*	Test	Δ*R*^2^	*p*
1. Cognitive Demographics Model				61.8	0.06	.097	–	–	–

Education, Years	0.58	0.18	.122	–	–	–	–	–	–

2. Self-Report Drug-Use Severity Model				63.3	0.08	.191	1 vs. 2	0.014	.457
Education, Years	0.60	0.19	.113	–	–	–	–	–	–
Regular Opioid Use, Years	0.26	0.05	.470	–	–	–	–	–	–

3. Self-Report Craving Model				65.0	0.08	.312	2 vs. 3	0.003	.611

Education, Years	0.60	0.18	.112	–	–	–	–	–	–
Regular Opioid Use, Years	0.24	0.05	.502	–	–	–	–	–	–
Picture Cue-Induced Craving	−0.16	0.30	.608	–	–	–	–	–	–

4. Drug-Biased Behavior Model				62.1	0.18	.075	3 vs. 4	0.102	.027∗
Education, Years	0.77	0.20	.061	–	–	–	–	–	–
Regular Opioid Use, Years	0.47	0.06	.290	–	–	–	–	–	–
Picture Cue-Induced Craving	−0.21	0.31	.533	–	–	–	–	–	–
Drug > Pleasant Explicit Choice	−0.75	0.00	.036∗	–	–	–	–	–	–

For each logistic regression, the standardized coefficient estimates (β), SEs, and *p* values are displayed for each predictor variable. Summary statistics (AIC, pseudo-*R*^2^, and *p* values) are displayed for each model vs. the null model. Increases in *R*^2^ values and *p* values are displayed for each step’s model comparison.

∗*p* < .05.

AIC, Akaike information criterion.

Similar results emerged from the regression using inpatient treatment dropout as the outcome measure, whereby the drug-biased behavior measure was the only regressor that significantly predicted treatment dropout in the final model (β = 0.81, *p* = .049) and the only measure that significantly improved the model’s predictive strength (Δ*R*^2^ = 0.11, *p* = .035) (see the Supplement). A post hoc regression with the behavioral measure alone approached significance (AIC = 51.9, pseudo-*R*^2^ = 0.11, *p* = .081).

For each outcome, post hoc hierarchical logistic regression analyses controlling for time in treatment yielded similar results (see the Supplement). Using factor scores instead of the highest-loading variables produced similar results for study treatment completion (see the Supplement). We also tested each unique pair of self-report and behavioral measures in 2-step hierarchical regressions across both outcomes: drug > pleasant explicit choice significantly improved prediction of inpatient treatment dropout when combined with regular opioid use (Δ*R*^2^ = 0.16, *p* = .048), with trends toward improving fit over other self-report measures (Δ*R*^2^ = 0.08−0.16, *p*s = .059–.099). Among self-report measures, only the Heroin Craving Questionnaire (HCQ) predicted outcomes, significantly explaining study treatment completion (*R*^2^ = 0.13, *p* = .032; null for inpatient dropout) (see the Supplement). Lastly, a sensitivity analysis excluding age from the factor analysis to interrogate a general cognitive factor yielded similar factor solution and regression results (see the Supplement).

## Discussion

Clinical research in addiction traditionally relies on self-report measures to characterize treatment end points, with limited success (53, 54, 55). Here, in inpatient iOUDs, we showed that an objective cognitive behavioral measure of drug bias, a proxy for drug seeking (39, 40, 41), assessed with a reliable and well-validated task that taps into a core addiction symptom, outperformed its self-reported (and cognitive demographic) counterparts in predicting treatment completion and dropout. First, in heroin-primary iOUDs, we replicated findings that had previously been demonstrated mostly in cocaine addiction (39,46) by showing drug-biased choice and fluency behavior. Compared with HC individuals, iOUDs selected more drug (and unpleasant) images and generated more drug words, supporting the generalizability of these measures as markers of drug bias in addiction; within participants, these behaviors were replicated at both baseline and follow-up. Following dimensionality reduction that yielded the hypothesized latent factors of cognitive demographics, self-report drug-use severity, self-report craving, and drug-biased behavior, we found that in iOUDs, only the drug-biased behavior measure (drug > pleasant explicit choice) significantly predicted study treatment completion in the full model. Importantly, the drug-biased behavior measure outperformed the self-report measures (regular opioid use and cue-induced craving) when cognitive demographics (years of education) was controlled for. Furthermore, the drug-biased behavioral measure again outperformed the self-report measures in predicting inpatient treatment dropout. Together, these novel results illustrate that disease-targeted, objectively assessed, cognitive behavioral measures are valuable markers of addiction severity, informing prospective treatment-related outcomes in iOUDs.

The drug-biased picture choice and verbal fluency results are consistent with biased attentional allocation toward drug stimuli in methadone-maintained outpatients with heroin addiction (56) and patients with opioid dependence and chronic pain (31,35). In general, this drug bias is consistent with an enhanced salience (encompassing attentional bias, reactivity, and subjective valence) attributed to drug over nondrug cues in people with addiction, which reinforces the cycle of addiction (7,8). Interestingly, despite the apparent drug bias in comparison with the control group, choice for drug images was the lowest of all image types in iOUDs, consistent with results in prescription opioid misuse (41), as well as other studies where addicted individuals rated drug cues as aversive (57,58) or even avoided drug cues in contexts where they are highly motivated to abstain (59). Additionally, we did not observe group-specific changes in drug-biased behavior with treatment; strong baseline-follow-up correlations suggest that these null effects reflect high test-retest reliability and/or sensitivity to stable, trait-like dimensions of addiction severity of these measures rather than state-related fluctuations. Future, larger studies could also inspect therapy group-specific recovery effects in these measures. Unlike previous reports in cocaine addiction (39), these results also revealed a bias toward unpleasant images, which is consistent with heightened emotional responses to unpleasant versus pleasant imagery in individuals with opioid dependence (60). This result may reflect frequent exposure to unpleasant stimuli or dysregulated affective processing in iOUDs (60,61), which remains to be explored. Additionally, iOUDs made a greater number of total image selections in the probabilistic choice task, indicating greater difficulty establishing deck preference relative to HC individuals. This may reflect impairments in self-awareness and insight (16,38) or approach-avoidance ambivalence toward drug cues in treatment-motivated patients.

For the first time in drug addiction, we have shown that compared with subjective self-report measures, an objective drug-biased behavioral measure can better account for future treatment completion and dropout, also objectively measured. The representative self-report measures (including cue-induced craving) did not significantly predict either outcome. This result stands in contrast to previous findings where craving predicted outcomes such as drug use and relapse (10,11,62), largely in studies involving actively using or early-abstinent populations and shorter follow-up intervals. Nevertheless, our results are consistent with previous reports of weak or null relationships of these self-report measures with relapse (63,64) and treatment dropout (65,66) across substances, with weaker craving-outcome associations reported specifically in iOUDs (11). A culprit may be the use of cross-sectional, static craving measurements, as evidence suggests that multiday, dynamic features of craving may better predict long-term outcomes (67). Notably, the emergence of the HCQ as predictive of outcome when tested in isolation may reflect the sensitivity of self-report scales to cognitive demographic factors, potentially masking their predictive value. This reconciles our null findings with previous reports of stronger self-report effects and underscores the advantage of behavioral measures in capturing outcome-relevant variance less influenced by such confounds.

The significant contribution of behavioral measures to outcome is consistent with our previous results where drug-biased picture choice predicted prospective (6 months) drug use in cocaine addiction (40) and prospective (8 weeks) opioid misuse severity in prescription opioid-treated pain patients (41). Another picture-viewing approach-avoidance task using drug-related images predicted 6-month cannabis use in heavy users, whereas self-report craving, motivation, and drug use did not (68). The predictive value of drug-biased behavioral measures may stem from their objective probing of conditioned responses to drug cues (e.g., attentional bias, physiological arousal) (69,70) shown to contribute to future relapse (34). Another contributing factor may be their greater reliance on higher-order cognitive functions (e.g., working memory, decision making) that are commonly impaired in addiction (7) as associated with poor treatment outcomes (30,71, 72, 73). We postulate that the modulation of these cognitive functions by a drug-related context confers the greatest predictive power because they exemplify the subversion of these functions toward the processing of drug-related cues, and at the expense of their nondrug counterparts, in people with addiction. Importantly, these results underscore the potential utility of drug-biased behavioral measures as actionable clinical tools for identifying high-risk individuals, enabling early, targeted interventions such as cue-bias modification and executive control training (e.g., drug cue reappraisal) to improve outcomes and mitigate treatment dropout.

Several limitations of the current study should be noted. Although the variables showing group differences (see Table 1) did not correlate with our outcome measures, samples more closely matched on demographics, smoking, and marijuana use could clarify their contribution. The modest *R*^2^ values suggest that additional latent factors may inform treatment outcomes, highlighting the need for larger samples to examine a wider range of self-report and behavioral measures, MOUD type/dose, therapy group-specific effects, and sex differences. Additionally, post hoc regressions with the behavioral measure alone approached significance in predicting outcomes, indicating that these measures should be used to supplement established self-report and cognitive demographic markers. Furthermore, while our selected outcomes can serve as important adjunct indicators of treatment status, further investigations are warranted to supplement these measures with other meaningful outcomes (e.g., abstinence duration, relapse severity) to align with FDA guidance (74). Lastly, whether these results generalize to other types of OUD, iOUDs receiving outpatient treatment, individuals not receiving MOUD, and/or high-risk populations earlier in treatment, remains to be tested, and future work extending these results to additional OUD samples would provide useful out-of-sample validation.

### Conclusions

There is an urgent need to develop and test measures that can be used to characterize drug-use severity and inform treatment outcomes to counteract the treatment-resistant nature of addiction. Here, we showed that choice and fluency tasks can serve as reliable markers of drug-biased behavior in iOUDs. Crucially, we demonstrated that an objective measure of drug-biased behavior outperformed traditional self-report measures in predicting treatment completion and dropout. Assessing a core deficit in drug addiction (i.e., attribution of salience toward drug over alternative cues) while circumventing the limitations of self-report and nonvarying categorical measures (e.g., drug toxicology), these objective cognitive behavioral measures of drug bias present viable alternatives and/or supplements to the outcome measures that have been most commonly utilized in clinical trials and other drug addiction research. Their use may lead to improved treatment models, allowing for early risk identification and the timely deployment of effective prevention efforts.

## Supplementary Material

Supplemental Text, Figures S1–S2, and Tables S1–S11

Key Resources Table
