# Supplementary material for: Moving Beyond Self-Report in Characterizing Drug Addiction: Using Drug-Biased Behavior to Predict Treatment Completion and Dropout in Heroin-Primary, Medication-Maintained Opioid Use Disorder
Source: Biol Psychiatry Glob Open Sci. 2025 Dec 9;6(2):100667. doi: 10.1016/j.bpsgos.2025.100667 (PMC12860340; doi:10.1016/j.bpsgos.2025.100667)
Supplement: Supplemental Text, Figures S1–S2, and Tables S1–S11 [file mmc1.pdf]

SUPPLEMENTARY INFORMATION

Moving Beyond Self-Report in Characterizing Drug Addiction: Using Drug-Biased Behavior to Predict Treatment Completion and Dropout in Heroin-Primary, Medication-Maintained Opioid Use Disorder

McClain*, Ceceli*, *et al.*

* These authors contributed equally to this work.

Supplemental Materials

Drug Treatment Program Details

All individuals with opioid use disorder (iOUD) were enrolled at an inpatient drug treatment program where they received relapse prevention treatment, anger management training, Seeking Safety therapy (a present-focused, cognitive-behavioral therapy model targeting trauma and/or addiction), as well as other counseling services.

Eligibility Criteria

All participants met the following inclusion criteria: 1) Ability to understand and give informed consent; and 2) 18-64 years of age. All iOUD further met the following inclusion criteria: 1) Diagnostic and Statistical Manual of Mental Disorders (DSM-5) diagnosis of OUD with heroin as the primary drug of choice; and 2) stabilized on medication for opioid use disorder (i.e., methadone or suboxone).

Participants were excluded from the study if they met any of the following criteria: 1) DSM-5 diagnosis for schizophrenia or developmental disorder (e.g., autism); 2) head trauma with loss of consciousness (>30 min); 3) history of neurological disease of central origin including seizures; 4) cardiovascular disease including high blood pressure and/or other medical conditions, including metabolic, endocrinological, oncological or autoimmune diseases, and infectious diseases common in iOUD (including Hepatitis B and C or HIV/AIDS); 5) metal implants or other MR contraindications (e.g., claustrophobia); and 6) women who were pregnant or lactating. To recruit a participant sample most representative of OUD in the real world, iOUD were not excluded for a DSM-5 diagnosis of a substance use disorder other than opiates, as long as opiates were the primary drug of choice and/or reason for treatment. Healthy controls (HC) were excluded if they met DSM-5 criteria for a substance use disorder; testing positive for drugs was also exclusionary.

Diagnostic Interviews, Psychiatric Diagnoses, and Comorbid Substance Use Disorders

A comprehensive diagnostic interview, encompassing the Mini International Neuropsychiatric Interview 7th ed. (1) and the Addiction Severity Index 5th ed. (2), was performed to assess DSM-5 criteria for major psychiatric and substance use disorders. In the iOUD, psychiatric diagnoses included major depressive disorder (n=29; 4 current), panic disorder (n=5; 3 current), agoraphobia (n=2; 1 current), obsessive compulsive disorder (n=1; 1 current), post-traumatic stress disorder (n=12; 6 current), binge eating disorder (n=1; 1 current), and generalized anxiety disorder (n=2; 2 current). Other substance use disorders included cocaine (n=20; 3 in sustained remission, 9 in early remission, 8 current) and other stimulants (n=1; in early remission), sedatives (n=10; 2 in sustained remission, 4 in early remission, 4 current), cannabis (n=3; all in early remission), and polysubstance use disorder (n=1; in sustained remission). In the HC group, the only psychiatric diagnosis was major depressive disorder (n=3; 0 current).

Assessments Presented in Table 1

At baseline, nicotine dependence was measured with the Fagerström Test for Nicotine Dependence (3); Depression and anxiety severity were measured using Beck's Depression (4) and Anxiety (5) Inventories, respectively; and heroin dependence, withdrawal, and craving were evaluated via the Severity of Dependence Scale (6), the Subjective Opiate Withdrawal Scale (7), and the Heroin Craving Questionnaire (HCQ, modified from Cocaine Craving Questionnaire (8)), respectively. Given the importance of craving for outcome prediction (9), we enriched its assessment by including the

following: drug wanting>liking (for the last drug use to intoxication) collected via the Sensitivity To Reinforcement of Addictive and Other Primary Rewards (STRAP-R) questionnaire (10); and picture cue- and movie scene-craving collected via self-report ratings of picture stimuli from an in-house drug cue-reactivity task (11) and clips from a drug-related movie (3-sec clips sampled every 30 seconds from the first 17-minutes of *Trainspotting* (12)).

Examining Changes in Drug-Biased Behavior

Longitudinal analyses of explicit choice (39 iOUD, 25 HC) revealed no main effect of group [$F(1,62)=0.11$, $p=0.737$], a significant main effect of cue type [pleasant>neutral>blank>negative>drug; $F(4,248) = 86.21$, $p<0.001$], no main effect of session [$F(1,62)=0.03$, $p=0.856$], a significant group*cue-type interaction [$F(4,248)=8.02$, $p<0.001$], no group*session [$F(1,62)=0.53$, $p=0.468$] or cue-type*session [$F(4,248)=1.25$, $p=0.292$] interaction, and no 3-way interaction [$F(4,248)=0.85$, $p=0.495$]. For probabilistic choice (38 iOUD, 24 HC), there was a main effect of group [iHUD>HC; $F(1,60)=7.54$, $p=0.008$] and cue-type [pleasant>neutral>unpleasant>drug; $F(3,180)=73.18$, $p<0.001$] but not session [$F(1,60)=0.46$, $p=0.500$], a significant group*cue-type interaction [$F(3,180)=4.56$, $p=0.004$], no group*session [$F(1,60)=0.79$, $p=0.379$] or cue-type*session [$F(3,180)=0.55$, $p=0.650$] interaction, and no 3-way interaction [$F(3,180)=0.77$, $p=0.510$]. For fluency (32 iOUD, 26 HC), there was no main effect of group [$F(1,56)=2.08$, $p=0.155$] or session [$F(1,56)=0.28$, $p=0.602$], a significant main effect of fluency type [nondrug>drug; $F(1,56) = 26.78$, $p<0.001$], a significant group*fluency-type interaction [$F(1,56)=17.06$, $p<0.001$], no group*session [$F(1,56)=1.70$, $p=0.197$] or session*fluency-type [$F(1,56)=2.59$, $p=0.113$] interaction, and no 3-way interaction [$F(1,56) = 1.16$, $p=0.286$] (see Figure S1). These results are largely consistent with those reported in the main text, where behavior showed no change between both administrations.

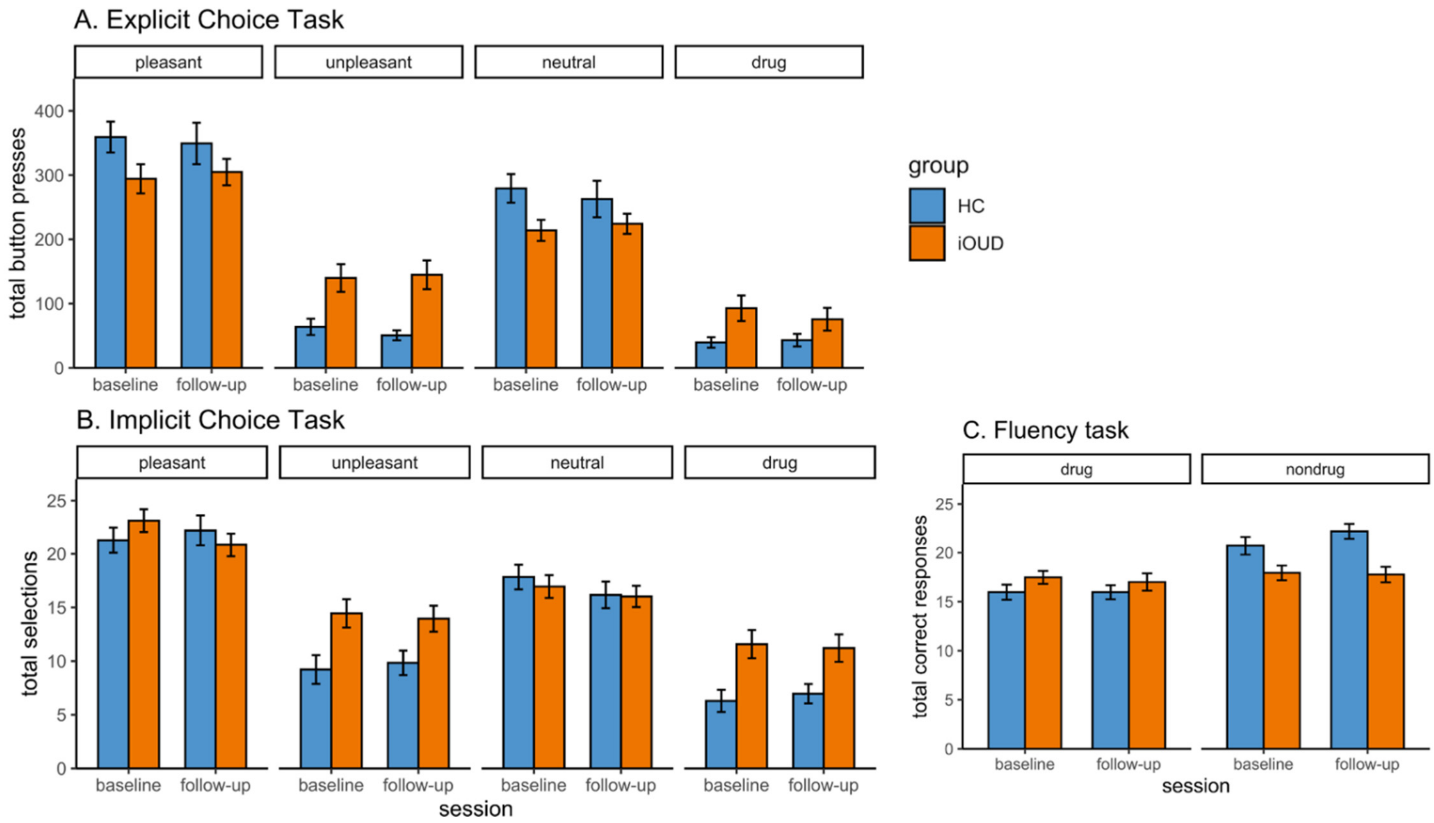

Figure S1. Longitudinal results in choice and fluency behavior. Individuals with opioid use disorder (iOUD) and healthy control (HC) participants' (A) explicit choice behavior, (B) probabilistic choice behavior, and C) fluency behavior revealing no reductions in drug-biased behaviors between baseline and follow-up in the subsets of participants with complete task data. Non-stimulus cues are not visualized. Error bars indicate SEM.

To further investigate task stability, we inspected correlations between baseline and follow-up performance within-subjects across tasks. Results revealed moderate to strong correlations (r>0.41, p<0.010), illustrating the high test-retest reliability that may be driving the null session-related effects (see Figure S2).

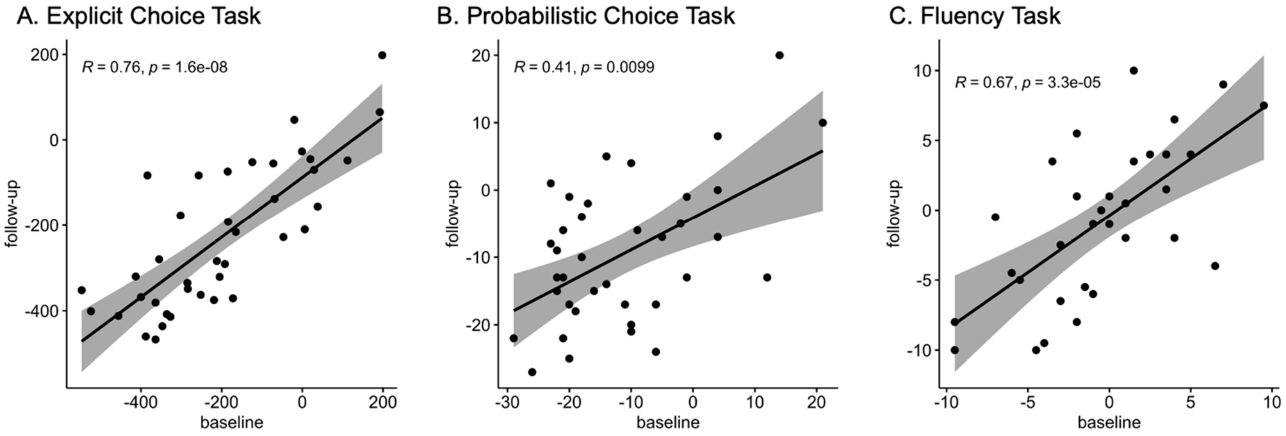

Figure S2. Intra-task correlations in choice and fluency behavior. Within-subject, intra-task correlations for (A) explicit choice (B) implicit choice and (C) fluency show positive correlations between baseline and follow-up behavior in the individuals with opioid use disorder (iOUD) with data at both timepoints.

Examining Potential Covariates in Baseline Analyses

To assess whether the group*cue-/fluency-type interaction effects survived corrections for potential covariates, the Table 1 measures that differed between the groups and showed significant associations (via Pearson or Spearman correlations) with our main behavioral measures (drug>pleasant explicit and probabilistic choice, and drug>non-drug fluency) [after correction for familywise error, α=.05/(7*3)=.003], were individually controlled for using group × cue-/fluency-type ANCOVAs. SOWS was included in these analyses to assess the effects of withdrawal, while smoking status was excluded due to its near parallel distribution between groups. Controlling for depression and anxiety (the only measures calling for these ANCOVAs) in the explicit choice model did not affect the results ($ps<0.001$); no corrections were needed for probabilistic choice. Correcting for depression in the fluency model also did not affect results (p=0.008).

Factor Analysis Details

Regarding the selection of variables to be included in the factor analysis, the continuous variables (see (13)) from the first three categories listed in Table 1 and the primary behavioral measures were included as they aligned with our constructs of interest (cognitive-demographics, self-report drug-use severity and craving, and drug-biased behavior); the other substance use, depression and anxiety measures in Table 1 were excluded, as they did not align with our hypothesized predictor categories. Additionally, past month heroin use was excluded due to its lack of variance. The remaining cognitive and demographics variables (age, education, verbal and nonverbal IQ) were intended to constitute a factor representing domains known to influence drug-biased behavioral task performance, serving as important non-drug neuropsychological covariates in the predictive models. The factor analysis was conducted using maximum likelihood estimation with varimax (orthogonal) rotation. Factor retention was determined by the Kaiser criterion (eigenvalues>1) and theoretical interpretability of the factor solution. For each factor, the raw values of the highest-loading variables, rather than the factor score estimates, were used in the primary regression models to enhance interpretability and generalizability, as clinicians would rely on the raw outputs rather than factor-derived composites. The factor analysis and regression models were conducted on the 52 iOUD who had complete data for all included measures (listed in Table 2 of the main text).

Demographics and Drug Use by Treatment Completion and Dropout

The study treatment completion iOUD subgroups did not differ in cognitive-demographics measures ($ps>0.043$), self-report drug use severity measures ($ps>0.122$), self-report craving measures ($ps>0.034$), other substance use variables ($ps>0.027$), depression and anxiety scores ($ps>0.922$), or drug-biased behavior ($ps>0.066$) after correcting for familywise error (α=.05/26=0.002). Similarly, inpatient treatment dropout subgroups did not differ in cognitive-demographics measures (ps>0.016), self-report drug use severity measures ($ps>0.213$), self-report craving ($ps>0.260$), other substance use variables ($ps>0.136$), depression and anxiety scores ($ps>0.831$), or drug-biased behavior ($ps>0.062$) after correcting for familywise error (0.002). See Table S1 for details on these measures between each set of iOUD subgroups.

	Study treatment completion			Inpatient treatment dropout		
	Attended (n=38)	Absent (n=14)	p-value	Non-dropout (n=42)	Dropout (n=10)	p-value
Demographics						
Age	42.50 (10.30)	37.39 (7.96)	0.083	42.73 (10.04)	34.41 (6.00)	0.016
Sex (Male/Female/Other)	28/9/1	12/2/0	0.610	32/9/1	8/2/0	0.878
Race (White/Black/Other)	27/5/6	13/0/1	0.218	30/5/7	10/0/0	0.156
Education (years)	12.37 (1.99)	11.36 (2.10)	0.043	12.17 (2.01)	11.80 (2.30)	0.445
Verbal IQ	96.18 (9.75)	94.00 (11.10)	0.493	96.24 (10.86)	92.90 (5.26)	0.168
Nonverbal IQ	9.79 (2.93)	8.71 (4.01)	0.295	9.40 (3.23)	9.90 (3.48)	0.669
Handedness (Right/Left)	30/8	12/2	0.879	33/9	9/1	0.706
Self-Report Drug Use Severity						
Regular Opioid Use (years)	11.38 (7.56)	10.04 (5.77)	0.733	11.51 (7.09)	8.95 (7.10)	0.213
Heroin Use Past Month (days)	0.32 (1.16)	0.00 (0.00)	0.122	0.29 (1.11)	0.00 (0.00)	0.216
Severity Of Dependence Scale (SDS)	11.24 (3.51)	12.14 (3.18)	0.358	11.48 (3.37)	11.50 (3.81)	0.878
Subjective Opiate Withdrawal Scale (SOWS)	3.39 (5.53)	3.00 (3.31)	0.871	3.33 (5.31)	3.10 (3.60)	0.884
Self-Report Craving						
Heroin Craving Questionnaire (HCQ)	38.79 (13.54)	48.93 (18.14)	0.034	40.33 (14.49)	46.50 (18.85)	0.260
Picture Cue-Induced Craving	1.98 (1.07)	2.17 (1.07)	0.419	2.02 (1.04)	2.07 (1.24)	0.926
Movie Scene-Induced Craving	1.18 (1.09)	1.08 (0.70)	0.820	1.21 (1.05)	0.90 (0.69)	0.585
STRAP-R	0.34 (1.42)	1.14 (1.92)	0.289	0.43 (1.47)	1.10 (2.02)	0.575
Other Substance Use						
Smoking Status (Current/Past/Never)	37/1/0	13/1/0	1.000[a]	40/2/0	10/0/0	1.000[a]
FTND	3.08 (1.89)[b]	4.38 (1.33)[c]	0.027	3.23 (1.93)[d]	4.20 (1.23)	0.136
Regular Marijuana Use (years)	8.18 (9.81)	8.75 (8.78)	0.668	8.55 (10.08)	7.45 (6.63)	0.925
Regular Alcohol Use (years)	7.95 (10.36)	6.82 (7.41)	0.890	7.67 (10.06)	7.55 (7.78)	0.792
Heroin Administration (Injection/Nasal/Oral/Smoking)	18/17/1/2	10/3/0/1	0.456	21/18/1/2	7/2/0/1	0.603
MOUD (Methadone/Suboxone)	34/4	11/3	0.573	38/4	7/3	0.234
Depression and Anxiety						
Beck's Depression Inventory (BDI)	14.45 (11.58)	15.85 (13.32)[e]	0.965	14.54 (11.11)[e]	15.90 (15.48)	0.831
Beck's Anxiety Inventory (BAI)	10.34 (8.80)	12.46 (14.16)[f]	0.922	10.32 (8.67)[f]	13.20 (15.77)	0.887
Drug-Biased Behavior						
Drug>pleasant explicit choice	-195.71 (186.75)	-92.86 (182.89)	0.066	-189.79 (187.23)	-76.60 (180.39)	0.062
Drug>pleasant probabilistic choice	-11.68 (12.15)	-6.50 (8.98)	0.066	-11.40 (7.63)	-5.60 (7.63)	0.081
Drug>nondrug fluency	0.17 (4.99)	1.04 (0.17)	0.524	0.01 (5.03)	2.30 (5.59)	0.211

Table S1. Sample profile at baseline for iOUD, by study treatment completion and inpatient treatment dropout subgroups. The measures are displayed for the same subset of iOUD used in the factor analysis and regression modeling (n=52 with complete data). To assess group differences across the continuous variables displayed in S1, t-tests were used for normally distributed variables, and Welch's t-test and Wilcoxon rank sum tests were used when assumptions of homogeneity of variance and normality were violated, respectively. Chi-square tests were used for unordered categorical and binary data comparisons. Significant between-group differences were corrected for familywise error (α=.05/26=.002). STRAP-R =Sensitivity to Reinforcement of Addictive and Other Primary Rewards; FTND=Fagerstorm Test for Nicotine Dependence; MOUD=medication for opioid use disorder.

[a]Smoking status "Never" was excluded from Chi-square test as it had no occurrences in either group.

[b,c]One missing FTND score.

[d]Two missing FTND scores.

[e]One missing BDI score.

[f]One missing BAI score.

Software Packages Used for Statistical Analyses

All statistical analyses were conducted in R (version 4.2.2). For the ANOVA tests, we used the *anova_test* function from the *rstatix* package, with assumptions of normality and homogeneity of variance evaluated using *shapiro.test* and *bartlett.test* from the base stats package. For post-hoc tests, we used the base R functions *t.test* and *wilcox.test.* Factor

analysis was performed using the *fa* function from the psych package. Hierarchical logistic regressions were conducted with the base R function *glm,* and model summary and comparison statistics were extracted with *summary* and *anova.* To evaluate model fit, we extracted pseudo-R^2 values using *r.squaredGLMM* from the *MuMIn* package and standardized regression parameters with the *standardize_parameters* function from the parameters package. All bar plots were produced using the *ggplot* function from the *ggplot2* package.

Hierarchical Logistic Regression with Inpatient Treatment Dropout

Hierarchical regression results with inpatient treatment dropout as the outcome revealed that the first model was not significant (R^2=0.01, p=0.603), such that cognitive-demographics (years of education) alone were not significantly associated with dropout (β=-0.19, p=0.609). The second model including the self-report drug-use severity measure (years of regular opioid use) was also not significant (R^2=0.04, p=0.462) and did not perform significantly better compared to cognitive-demographics alone (ΔR^2=0.032, p=0.259). Similarly, the third model with self-report craving (picture cue-induced craving) was not significant (R^2=0.04, p=0.671) and did not perform significantly better compared to the self-report drug-use severity model (ΔR^2<0.001, p=0.970). However, adding the drug-biased behavior measure (drug>pleasant explicit choice) significantly increased the variability in inpatient treatment dropout explained by the model, increasing the pseudo-R^2 by 11.3% (p=0.035). In this final model, the drug-biased behavior measure was significantly associated with the outcome such that the higher the baseline drug-biased behavior, the higher the likelihood of treatment dropout (β=0.81, p=0.049), not seen for the demographic, self-report drug-use severity and self-report craving measures (ps>0.170). In the final model, 15% of the variance in the likelihood of treatment dropout was associated with the model regressors (p=0.201; Table S2).

				Model summary			Model Comparison		
	β	SE	p-value	AIC	R^2	p-value	Test	ΔR^2	p-value
1. Cognitive-Demographics Model				54.6	0.01	0.603	--	--	--
Education (years)	-0.19	0.18	0.609						
2. Self-Report Drug-Use Severity Model				55.3	0.04	0.462	1 vs. 2	0.032	0.259
Education (years)	-0.23	0.19	0.552						
Regular opioid use (years)	-0.46	0.06	0.291						
3. Self-Report Craving Model				57.4	0.04	0.671	2 vs. 3	0.000	0.970
Education (years)	-0.23	0.19	0.552						
Regular opioid use (years)	-0.45	0.06	0.293						
Picture cue-induced craving	0.01	0.34	0.969						
4. Objective Model				54.9	0.15	0.201	3 vs. 4	0.113	**0.035**
Education (years)	-0.38	0.20	0.364						
Regular opioid use (years)	-0.78	0.08	0.170						
Picture cue-induced craving	0.07	0.35	0.855						
Drug>pleasant explicit choice	0.81	0.00	**0.049**						

Table S2. Hierarchical regression analysis of predictors of inpatient treatment dropout. Hierarchical logistic regression results are shown for the 52 individuals with opioids use disorder (iOUD) included in the factor analysis. For each logistic regression, the standardized coefficient estimates (β), standard errors (SE), and p-values are displayed for each predictor variable. Summary statistics (AIC, pseudo-R^2, and p-values) are displayed for each model vs. the null model. Increases in R^2 values and p-values are displayed for each step's model comparison. P-values below 0.05 are denoted in bold typeface.

Hierarchical Logistic Regressions Controlling for Time in Treatment

Given our sample represented iOUD at various stages of treatment, we performed a post-hoc analysis whereby time in treatment (days; at baseline) was controlled for at every step of the hierarchical logistic regression.

Results for study treatment completion revealed that controlling for time in treatment in the hierarchical logistic regression yielded similar results whereby the drug-biased behavior measure was the only regressor that significantly predicted treatment dropout in the final model (β=-0.75, p=0.037), and the only measure that significantly improved the model's predictive strength (ΔR^2=0.10, p=0.028; Table S3). The same analysis for inpatient treatment dropout revealed that the drug-biased behavior measure was the only regressor that approached significance in the final model (β=0.81, p=0.050), and the only measure that significantly improved the model's predictive strength (ΔR^2=0.18, p=0.037; Table S4).

				Model summary			Model Comparison		
	β	SE	p-value	AIC	R^2	p-value	Test	ΔR^2	p-value
1. Cognitive-Demographics Model				63.5	0.14	0.594	--	--	--
Education (years)	0.51	0.19	0.186						
Time in treatment (days)	0.60	0.00	0.741						
2. Self-Report Drug-Use Severity Model				65.1	0.19	0.695	1 vs. 2	-0.013	0.505
Education (years)	0.55	0.19	0.163						
Regular opioid use (years)	0.23	0.05	0.516						
Time in treatment (days)	0.45	0.00	0.776						
3. Self-Report Craving Model				66.9	0.16	0.811	2 vs. 3	-0.026	0.629
Education (years)	0.56	0.19	0.157						
Regular opioid use (years)	0.22	0.05	0.547						
Picture cue-induced craving	-0.16	0.30	0.625						
Time in treatment (days)	0.34	0.00	0.783						
4. Drug-Biased Behavior Model				64.0	0.29	0.214	3 vs. 4	0.100	**0.028**
Education (years)	0.74	0.21	0.081						
Regular opioid use (years)	0.45	0.06	0.310						
Picture cue-induced craving	-0.20	0.32	0.564						
Drug>pleasant explicit choice	-0.75	0.00	**0.037**						
Time in treatment (days)	0.31	0.00	0.832						

Table S3. Hierarchical regression analysis of predictors of study treatment completion controlling for time in treatment. Hierarchical logistic regression results are shown for the 52 individuals with opioids use disorder (iOUD) included in the factor analysis. For each logistic regression, the standardized coefficient estimates (β), standard errors (SE), and p-values are displayed for each predictor variable. Summary statistics (AIC, pseudo-R^2, and p-values) are displayed for each model vs. the null model. Increases in R^2 values and p-values are displayed for each step's model comparison. P-values below 0.05 are denoted in bold typeface.

	β	SE	p-value	Model summary AIC	R²	p-value	Model Comparison Test	ΔR²	p-value
1. Cognitive-Demographics Model				56.4	0.07	0.595	--	--	--
Education (years)	-0.13	0.19	0.738						
Time in treatment (days)	-0.43	0.00	0.734						
2. Self-Report Drug-Use Severity Model				57.3	0.10	0.501	1 vs. 2	0.031	0.295
Education (years)	-0.19	0.19	0.631						
Regular opioid use (years)	-0.27	0.06	0.327						
Time in treatment (days)	-0.43	0.00	0.803						
3. Self-Report Craving Model				59.3	0.10	0.710	2 vs. 3	-0.000	0.977
Education (years)	-0.19	0.19	0.630						
Regular opioid use (years)	-0.43	0.06	0.328						
Picture cue-induced craving	0.01	0.34	0.977						
Time in treatment (days)	-0.26	0.00	0.804						
4. Drug-Biased Behavior Model				56.9	0.27	0.220	3 vs. 4	0.175	**0.037**
Education (years)	-0.36	0.21	0.404						
Regular opioid use (years)	-0.76	0.08	0.184						
Picture cue-induced craving	0.06	0.35	0.875						
Drug>pleasant explicit choice	0.81	0.00	0.050						
Time in treatment (days)	-0.19	0.00	0.875						

Table S4. Hierarchical regression analysis of predictors of inpatient treatment dropout controlling for time in treatment. Hierarchical logistic regression results are shown for the 52 individuals with opioids use disorder (iOUD) included in the factor analysis. For each logistic regression, the standardized coefficient estimates (β), standard errors (SE), and p-values are displayed for each predictor variable. Summary statistics (AIC, pseudo-R^2, and p-values) are displayed for each model vs. the null model. Increases in R^2 values and p-values are displayed for each step's model comparison. P-values below 0.05 are denoted in bold typeface.

Hierarchical Logistic Regressions with Factor Score Regressors

To evaluate the relationship between all drug-biased behavior tasks and our outcome measures, we reran the hierarchical logistic regression models using the factor score estimates as the regressors in place of individual variables.

Results revealed that the first model with the cognitive-demographics factor scores alone significantly improved fit compared to the intercept-only model in predicting study treatment completion (R^2=0.12, p=0.042), but not inpatient treatment dropout (R^2=0.04, p=0.281). The second model including the self-report drug-use severity factor scores was not significant (R^2s<0.15, ps>0.095) and did not perform significantly better compared to cognitive-demographics factor scores alone (ΔR^2s<0.08, ps>0.222) for either outcome. Similarly, the third model with the self-report craving factor scores was not significant (R^2s<0.16, ps>0.152) and did not perform significantly better compared to the self-report drug-use severity model (ΔR^2s<0.02, ps>0.447) for either outcome. However, adding the drug-biased behavior factor scores significantly increased the variability in study treatment completion explained by the model, corresponding to a 12% improvement in variance accounted for by the model (p=0.037)—with a similar trend for inpatient treatment dropout (ΔR^2=0.13, p=0.061). In the final models, greater baseline drug-biased responding across tasks was associated with a lower likelihood of study treatment completion (β=-0.68, p=0.047)—with a similar trend for inpatient treatment dropout (β=0.68, p=0.075). The cognitive-demographics factor was also significant such that its higher expression was associated with a higher likelihood of study treatment completion (β=0.83, p=0.039), not observed for the self-report drug-use severity and self-report craving factor regressors (ps>0.403). In the final models, 28% of the variance in the likelihood of treatment completion (p=0.047)

and 25% of the variance in the likelihood of inpatient treatment dropout (*p*=0.186) were associated with the model regressors (Tables S5 and S6).

				Model summary			Model Comparison		
	β	SE	p-value	AIC	R^2	p-value	Test	ΔR^2	p-value
1. Cognitive-Demographics Model				60.3	0.12	**0.042**	--	--	--
Cognitive-Demographics Factor	0.68	0.46	0.056						
2. Self-Report Severity Model				61.9	0.15	0.095	1 vs. 2	0.02	0.453
Cognitive-Demographics Factor	0.69	0.46	0.057						
Self-Report Drug-Use Severity Factor	0.27	0.37	0.468						
3. Self-Report Craving Model				63.3	0.16	0.152	2 vs. 3	0.02	0.447
Cognitive-Demographics Factor	0.72	0.47	**0.050**						
Self-Report Drug-Use Severity Factor	0.26	0.37	0.474						
Self-Report Craving Factor	-0.26	0.34	0.445						
4. Drug-Biased Behavior Model				60.9	0.28	**0.047**	3 vs. 4	0.12	**0.037**
Cognitive-Demographics Factor	0.83	0.52	**0.039**						
Self-Report Drug-Use Severity Factor	0.34	0.42	0.409						
Self-Report Craving Factor	-0.29	0.35	0.403						
Drug-Biased Behavior Factor	-0.68	0.35	**0.047**						

Table S5. Hierarchical regression analysis of predictors of study treatment completion with factor score estimates as regressors. Hierarchical logistic regression results are shown for the 52 individuals with opioids use disorder (iOUD) included in the factor analysis. For each logistic regression, the standardized coefficient estimates (β), standard errors (SE), and p-values are displayed for each predictor variable. Summary statistics (AIC, pseudo-R^2, and p-values) are displayed for each model vs. the null model. Increases in R^2 values and p-values are displayed for each step's model comparison. P-values below 0.05 are denoted in bold typeface.

				Model summary			Model Comparison		
	β	SE	p-value	AIC	R^2	p-value	Test	ΔR^2	p-value
1. Cognitive-Demographics Model				53.8	0.04	0.281	--	--	--
Cognitive-Demographics Factor	-0.39	0.47	0.289						
2. Self-Report Severity Model				54.3	0.12	0.265	1 vs. 2	0.08	0.222
Cognitive-Demographics Factor	-0.40	0.49	0.294						
Self-Report Drug-Use Severity Factor	-0.52	0.46	0.260						
3. Self-Report Craving Model				56.2	0.12	0.446	2 vs. 3	0.00	0.923
Cognitive-Demographics Factor	-0.40	0.49	0.300						
Self-Report Drug-Use Severity Factor	-0.52	0.46	0.260						
Self-Report Craving Factor	-0.04	0.37	0.923						
4. Drug-Biased Behavior Model				54.7	0.25	0.186	3 vs. 4	0.13	0.061
Cognitive-Demographics Factor	-0.46	0.54	0.285						
Self-Report Drug-Use Severity Factor	-0.63	0.52	0.223						
Self-Report Craving Factor	-0.02	0.38	0.962						
Drug-Biased Behavior Factor	0.68	0.39	0.075						

Table S6. Hierarchical regression analysis of predictors of inpatient treatment dropout with factor score estimates as regressors. Hierarchical logistic regression results are shown for the 52 individuals with opioids use disorder (iOUD) included in the factor analysis. For each logistic regression, the standardized coefficient estimates (β), standard errors (SE), and p-values are displayed for each predictor variable. Summary statistics (AIC, pseudo-R^2, and p-values) are displayed for each model vs. the null model. Increases in R^2 values and p-values are displayed for each step's model comparison. P-values below 0.05 are denoted in bold typeface.

Two-Step Hierarchical Regression Models of Self-Report and Drug-Biased Behavior Measures

To explore direct comparisons between each self-report and drug-biased behavioral measure, we conducted two-step hierarchical logistic regressions (self-report in step 1, drug-biased behavior in step 2) for every unique combination of self-

report and behavior measures, for both study treatment completion and inpatient treatment dropout outcomes (see Tables S7 and S8).

These analyses revealed that, similar to our main results, drug>pleasant explicit choice significantly improved the model with regular opioid use (years; ΔR^2=0.16, p=0.048) in predicting inpatient treatment dropout—with a similar trend for study treatment completion (ΔR^2=0.11, p=0.059). In the two-predictor models, drug>pleasant explicit choice approached significance across both outcomes ($|\beta|$s>0.63, ps<0.071) while years of regular opioid use did not ($|\beta|$s<0.65, ps>0.197). There was a consistent trend for the drug>pleasant explicit choice task to improve predictive strength when added after the self-report step (ΔR^2s=0.08–0.16, ps=0.059–0.099), including the representative self-report craving measure (picture cue-induced craving; $\Delta R^2 s$>0.10, ps<0.082), across both outcomes. Although drug>pleasant probabilistic choice trended towards significantly improving the models with regular opioid use for both outcomes (ΔR^2s=0.09-0.14, ps=0.063-0.092), drug>pleasant probabilistic choice and drug>nondrug fluency did not significantly account for additional variance above and beyond self-report measures (ΔR^2s<0.07, ps>0.125); nor were they significant predictors in any of the models ($|\beta|$s<0.72, ps>0.073), across both outcomes.

None of the self-report measures accounted for a significant proportion of variance in treatment outcomes, with the exception of the Heroin Craving Questionnaire (HCQ), which predicted study treatment completion both when entered alone (R^2=0.13, p=0.032) and when combined with drug>pleasant explicit choice (R^2=0.23, p=0.018); however, these associations did not extend to inpatient treatment dropout (R^2=0.05, p=0.255 and R^2=0.16, p=0.110, respectively). Importantly, when paired with HCQ, drug>pleasant explicit choice approached significance in the two-regressor model (β=-0.63, p=0.077) and in improving the model's predictive strength (ΔR^2=0.10, p=0.065), suggesting it may explain a unique portion of variance in study treatment completion above and beyond that accounted for by craving.

				Model summary			Model Comparison		
	β	SE	p-value	AIC	R^2	p-value	Test	ΔR^2	p-value
Model 1				64.2	0.01	0.535	--	--	--
Regular opioid use (years)	0.20	0.05	0.544						
Model 2				62.6	0.12	0.140	1 vs. 2	0.11	0.059
Regular opioid use (years)	0.32	0.05	0.396						
Drug>pleasant explicit choice	-0.63	0.00	0.071						
Model 3				63.3	0.10	0.200	1 vs. 3	0.09	0.092
Regular opioid use (years)	0.39	0.05	0.304						
Drug>pleasant probabilistic choice	-0.57	0.03	0.102						
Model 4				65.8	0.02	0.684	1 vs. 4	0.01	0.540
Regular opioid use (years)	0.19	0.05	0.568						
Drug>nondrug fluency	-0.19	0.06	0.540						
Model 5				63.8	0.03	0.379	--	--	--
Severity of Dependence Scale	-0.29	0.10	0.397						
Model 6				52.6	0.12	0.135	5 vs. 6	0.10	0.072
Severity of Dependence Scale	-0.32	0.11	0.375						
Drug>pleasant explicit choice	-0.60	0.00	0.085						
Model 7				63.7	0.09	0.235	5 vs. 7	0.06	0.145
Severity of Dependence Scale	-0.32	0.11	0.384						
Drug>pleasant probabilistic choice	-0.46	0.03	0.149						
Model 8				65.5	0.04	0.58	5 vs. 8	0.01	0.584
Severity of Dependence Scale	-0.28	0.10	0.434						
Drug>nondrug fluency	-0.18	0.06	0.583						
Model 9				64.5	0.00	0.794	--	--	--
Subjective Opiate Withdrawal Scale	0.09	0.07	0.799						
Model 10				63.4	0.09	0.206	9 vs. 10	0.09	0.078
Subjective Opiate Withdrawal Scale	0.03	0.07	0.922						
Drug>pleasant explicit choice	-0.58	0.00	0.091						
Model 11				64.4	0.06	0.336	9 vs. 11	0.06	0.146
Subjective Opiate Withdrawal Scale	0.11	0.07	0.748						
Drug>pleasant probabilistic choice	-0.46	0.03	0.152						
Model 12				66.1	0.01	0.801	9 vs. 12	0.01	0.540
Subjective Opiate Withdrawal Scale	0.05	0.07	0.882						
Drug>nondrug fluency	-0.19	0.06	0.540						
Model 13				60.0	0.13	**0.032**	--	--	--
Heroin Craving Questionnaire	-0.71	0.02	**0.045**						
Model 14				58.6	0.23	**0.018**	13 vs. 14	0.10	0.065
Heroin Craving Questionnaire	-0.76	0.02	**0.042**						
Drug>pleasant explicit choice	-0.63	0.00	0.077						
Model 15				60.6	0.18	0.050	13 vs. 15	0.04	0.235
Heroin Craving Questionnaire	-0.68	0.02	0.064						
Drug>pleasant probabilistic choice	-0.39	0.03	0.238						
Model 16				61.7	0.15	0.085	13 vs. 16	0.02	0.556
Heroin Craving Questionnaire	-0.71	0.02	**0.048**						
Drug>nondrug fluency	-0.19	0.06	0.556						
Model 17				64.3	0.00	0.581	--	--	--
Picture Cue-Induced Craving	-0.17	0.29	0.578						
Model 18				63.1	0.10	0.174	17 vs. 18	0.10	0.074
Picture Cue-Induced Craving	-0.18	0.29	0.559						
Drug>pleasant explicit choice	-0.59	0.00	0.086						
Model 19				64.2	0.07	0.311	17 vs. 19	0.06	0.578
Picture Cue-Induced Craving	-0.17	0.30	0.602						
Drug>pleasant probabilistic choice	-0.45	0.03	0.160						
Model 20				65.7	0.03	0.645	17 vs. 20	0.02	0.450
Picture Cue-Induced Craving	-0.21	0.29	0.497						
Drug>nondrug fluency	-0.24	0.06	0.451						
Model 21				64.5	0.00	0.744	--	--	--
Movie Scene-Induced Craving	0.10	0.33	0.747						
Model 22				63.4	0.10	0.205	21 vs. 22	0.10	0.080
Movie Scene-Induced Craving	0.89	0.33	0.893						
Drug>pleasant explicit choice	0.09	0.00	0.092						
Model 23				64.2	0.04	0.311	21 vs. 23	0.06	0.135
Movie Scene-Induced Craving	0.16	0.33	0.612						
Drug>pleasant probabilistic choice	-0.47	0.03	0.143						

Model 24				66.1	0.01	0.787	21 vs. 24	0.01	0.542
Movie Scene-Induced Craving	0.08	0.33	0.812						
Drug>nondrug fluency	-0.19	0.06	0.543						
Model 25				62.0	0.08	0.105	--	--	--
STRAP-R	-0.51	0.20	0.112						
Model 26				61.1	0.16	0.065	25 vs. 26	0.08	0.093
STRAP-R	-0.49	0.21	0.138						
Drug>pleasant explicit choice	-0.58	0.00	0.106						
Model 26				62.3	0.12	0.117	25 vs. 27	0.05	0.198
STRAP-R	-0.49	0.21	0.143						
Drug>pleasant probabilistic choice	-0.41	0.03	0.201						
Model 26				63.8	0.08	0.250	25 vs. 28	0.00	0.698
STRAP-R	-0.49	0.21	0.133						
Drug>nondrug fluency	-0.12	0.06	0.697						

Table S7. Two-step hierarchical regression analyses of predictors of study treatment completion. Hierarchical logistic regression results are shown for the 52 individuals with opioids use disorder (iOUD) included in the factor analysis. For each logistic regression, the standardized coefficient estimates (β), standard errors (SE), and p-values are displayed for each predictor variable. Summary statistics (AIC, pseudo-R^2, and p-values) are displayed for each model vs. the null model. Increases in R^2 values and p-values are displayed for each step's model comparison. P-values below 0.05 are denoted in bold typeface. STRAP-R=Sensitivity to Reinforcement of Addictive and Other Primary Rewards.

				Model summary			Model Comparison		
	β	SE	p-value	AIC	R^2	p-value	Test	ΔR^2	p-value
Model 1				53.7	0.05	0.278	--	--	--
Regular opioid use (years)	-0.43	0.06	0.308						
Model 2				51.8	0.21	0.079	1 vs. 2	0.16	**0.048**
Regular opioid use (years)	-0.65	0.07	0.197						
Drug>pleasant explicit choice	0.76	0.00	0.063						
Model 3				52.3	0.19	0.099	1 vs. 3	0.14	0.063
Regular opioid use (years)	-0.72	0.07	0.147						
Drug>pleasant probabilistic choice	0.72	0.03	0.073						
Model 4				54.3	0.11	0.267	1 vs. 4	0.05	0.226
Regular opioid use (years)	-0.43	0.06	0.330						
Drug>nondrug fluency	0.42	0.07	0.230						
Model 5				54.9	0.00	0.984	--	--	--
Severity of Dependence Scale	0.01	0.10	0.984						
Model 6				53.9	0.11	0.219	5 vs. 6	0.11	0.082
Severity of Dependence Scale	0.01	0.11	0.971						
Drug>pleasant explicit choice	0.65	0.00	0.097						
Model 7				54.9	0.07	0.365	5 vs. 7	0.07	0.155
Severity of Dependence Scale	0.01	0.11	0.989						
Drug>pleasant probabilistic choice	0.50	0.03	0.159						
Model 8				55.3	0.05	0.452	5 vs. 8	0.05	0.208
Severity of Dependence Scale	-0.05	0.11	0.901						
Drug>nondrug fluency	0.44	0.07	0.210						
Model 9				54.9	0.00	0.892	--	--	--
Subjective Opiate Withdrawal Scale	-0.05	0.07	0.894						
Model 10				53.9	0.11	0.220	9 vs. 10	0.11	0.083
Subjective Opiate Withdrawal Scale	0.01	0.08	0.930						
Drug>pleasant explicit choice	0.65	0.00	0.098						
Model 11				54.9	0.07	0.358	9 vs. 11	0.07	0.153
Subjective Opiate Withdrawal Scale	-0.07	0.07	0.850						
Drug>pleasant probabilistic choice	0.50	0.03	0.157						
Model 12				55.3	0.06	0.453	9 vs. 12	0.05	0.211
Subjective Opiate Withdrawal Scale	0.03	0.07	0.930						
Drug>nondrug fluency	0.44	0.07	0.216						
Model 13				53.6	0.05	0.255	--	--	--
Heroin Craving Questionnaire	0.40	0.02	0.259						
Model 14				52.5	0.16	0.110	13 vs. 14	0.12	0.078
Heroin Craving Questionnaire	0.43	0.02	0.246						
Drug>pleasant explicit choice	0.67	0.00	0.095						
Model 15				54.0	0.10	0.234	13 vs. 15	0.06	0.204
Heroin Craving Questionnaire	0.34	0.02	0.350						
Drug>pleasant probabilistic choice	0.45	0.03	0.206						
Model 16				54.1	0.10	0.251	13 vs. 16	0.05	0.226
Heroin Craving Questionnaire	0.40	0.02	0.279						
Drug>nondrug fluency	0.43	0.07	0.229						
Model 17				54.9	0.00	0.908	--	--	--
Picture Cue-Induced Craving	0.04	0.33	0.908						
Model 18				53.9	0.11	0.218	17 vs. 18	0.11	0.082
Picture Cue-Induced Craving	0.05	0.33	0.892						
Drug>pleasant explicit choice	0.65	0.00	0.097						
Model 19				54.9	0.07	0.363	17 vs. 19	0.07	0.156
Picture Cue-Induced Craving	0.03	0.35	0.942						
Drug>pleasant probabilistic choice	0.49	0.03	0.160						
Model 20				55.2	0.06	0.425	17 vs. 20	0.03	0.192
Picture Cue-Induced Craving	0.14	0.34	0.706						
Drug>nondrug fluency	0.47	0.07	0.200						
Model 21				54.0	0.04	0.348	--	--	--
Movie Scene-Induced Craving	-0.36	0.41	0.371						
Model 22				53.3	0.14	0.166	21 vs. 22	0.10	0.099
Movie Scene-Induced Craving	-0.29	0.40	0.472						
Drug>pleasant explicit choice	0.63	0.00	0.114						
Model 23				53.7	0.11	0.199	21 vs. 23	0.07	0.125
Movie Scene-Induced Craving	-0.42	0.41	0.299						
Drug>pleasant probabilistic choice	0.54	0.03	0.132						

Model 24				54.8	0.07	0.345	21 vs. 24	0.04	0.264
Movie Scene-Induced Craving	-0.30	0.42	0.471						
Drug>nondrug fluency	0.39	0.07	0.272						
Model 25				53.5	0.05	0.231	--	--	--
STRAP-R	0.42	0.22	0.231						
Model 26				52.7	0.15	0.122	25 vs. 26	0.10	0.096
STRAP-R	0.38	0.22	0.280						
Drug>pleasant explicit choice	0.64	0.00	0.113						
Model 26				53.8	0.10	0.212	25 vs. 27	0.05	0.196
STRAP-R	0.37	0.23	0.297						
Drug>pleasant probabilistic choice	0.45	0.03	0.199						
Model 26				54.3	0.09	0.270	25 vs. 28	0.04	0.277
STRAP-R	0.36	0.22	0.308						
Drug>nondrug fluency	0.39	0.07	0.275						

Table S8. Two-step hierarchical regression analyses of predictors of inpatient treatment dropout. Hierarchical logistic regression results are shown for the 52 individuals with opioids use disorder (iOUD) included in the factor analysis. For each logistic regression, the standardized coefficient estimates (β), standard errors (SE), and p-values are displayed for each predictor variable. Summary statistics (AIC, pseudo-R^2, and p-values) are displayed for each model vs. the null model. Increases in R^2 values and p-values are displayed for each step's model comparison. P-values below 0.05 are denoted in bold typeface. STRAP-R=Sensitivity to Reinforcement of Addictive and Other Primary Rewards.

Sensitivity Analysis: Exploring a General Cognitive Factor

Given that age represents a demographic characteristic, whereas years of education and IQ scores index cognitive and neuropsychological functioning, we conducted a sensitivity analysis in which age was excluded from the factor analysis. This exclusion was hypothesized to yield a more cohesive general cognitive factor. The factor analysis results revealed four factors consistent with our *a priori* hypothesized constructs, with verbal IQ (λ=0.53) representing the general cognitive functioning factor in place of years of education. The other highest-loading variables remained the same as the main results: years of regular opioid use (self-report drug-use severity; λ=0.88); picture cue-induced craving (self-report craving; λ=0.94); and drug>pleasant explicit choice (drug-biased behavior; λ=0.99) (Table S9).

	Factor Loadings					
	1	2	3	4	h^2	$1-h^2$
Cognitive Functioning						
Education (years)	0.11	-0.10	-0.13	0.51	0.30	0.70
Verbal IQ	0.16	0.02	0.01	**0.53**	0.31	0.69
Nonverbal IQ	-0.19	0.04	0.08	0.45	0.25	0.75
Self-Report Drug-Use Severity						
Regular opioid use (years)	0.10	0.14	**0.88**	0.14	0.82	0.18
SDS	0.01	0.09	0.00	-0.11	0.02	0.98
SOWS	-0.11	-0.03	0.35	-0.04	0.14	0.86
Self-Report Craving						
HCQ	0.03	0.35	0.08	-0.22	0.18	0.82
Picture cue-induced craving	-0.01	**0.94**	-0.29	0.16	1.00	0.01
Movie scene-induced craving	-0.11	0.54	-0.01	-0.04	0.31	0.69
STRAP-R	0.08	-0.12	0.45	-0.06	0.23	0.78
Drug-Biased Behavior						
Drug>pleasant explicit choice	**0.99**	-0.02	0.01	0.08	1.00	0.01
Drug>pleasant probabilistic choice	0.51	0.19	0.26	-0.32	0.46	0.54
Drug>nondrug fluency	0.42	-0.21	-0.10	0.13	0.25	0.75
Eigenvalue	1.53	1.44	1.28	0.98		
% of variance	29%	27%	24%	19%		
Cumulative %	29%	57%	81%	100%		

Table S9. Factor analysis results with age excluded. Factor loadings, communality (h^2), uniqueness ($1-h^2$), eigenvalues, percentages of variance and cumulative percentages of variance are shown. The extraction method used was maximum likelihood estimation with a varimax rotation method. Conducted in 52 individuals with opioid use disorder (iOUD) with complete data for all variables. SDS=Severity of Dependence Scale; SOWS=Subjective Opiate Withdrawal Scale; HCQ=Heroin Craving Questionnaire; STRAP-R=Sensitivity to Reinforcement of Addictive and Other Primary Rewards.

Using this new cognitive-demographics regressor in place of years of education revealed similar effects as observed in the main results, whereby drug>pleasant explicit choice was the only variable to significantly predict study treatment completion and inpatient treatment dropout ($|\beta|$s>0.69, ps<0.049) and the only measure to significantly improve the model's predictive strength ($\Delta R^2>0.091$, ps<0.040; Table S10 and S11).

	β	SE	p-value	Model summary AIC	R^2	p-value	Model Comparison Test	ΔR^2	p-value
1. Cognitive Functioning Model				64.1	0.01	0.483	--	--	--
Verbal IQ	0.22	0.03	0.486						
2. Self-Report Drug-Use Severity Model				65.8	0.02	0.667	1 vs. 2	0.010	0.573
Verbal IQ	0.21	0.03	0.517						
Regular opioid use (years)	0.18	0.05	0.581						
3. Self-Report Craving Model				67.4	0.02	0.766	2 vs. 3	0.010	0.562
Verbal IQ	0.23	0.03	0.477						
Regular opioid use (years)	0.17	0.05	0.623						
Picture cue-induced craving	-0.18	0.29	0.559						
4. Drug-Biased Behavior Model				65.2	0.15	0.252	3 vs. 4	0.117	**0.040**
Verbal IQ	0.37	0.03	0.298						
Regular opioid use (years)	0.27	0.05	0.488						
Picture cue-induced craving	-0.22	0.31	0.493						
Drug>pleasant explicit choice	-0.69	0.00	**0.049**						

Table S10. Hierarchical logistic regression analysis for study treatment completion. For each logistic regression, the standardized coefficient estimates (β), standard errors (SE), and p-values are displayed for each predictor variable. Summary statistics (AIC, pseudo-R^2, and p-values) are displayed for each model vs. the null model. Increases in R^2 values and p-values are displayed for each step's model comparison. P-values below 0.05 are denoted in bold typeface.

	β	SE	p-value	Model summary AIC	R^2	p-value	Model Comparison Test	ΔR^2	p-value
1. Cognitive Functioning Model				54.0	0.03	0.341	--	--	--
Verbal IQ	-0.34	0.04	0.346						
2. Self-Report Drug-Use Severity Model				55.0	0.08	0.385	1 vs. 2	0.042	0.316
Verbal IQ	-0.31	0.04	0.395						
Regular opioid use (years)	-0.39	0.06	0.344						
3. Self-Report Craving Model				57.0	0.08	0.587	2 vs. 3	0.000	0.896
Verbal IQ	-0.32	0.04	0.391						
Regular opioid use (years)	-0.39	0.06	0.350						
Picture cue-induced craving	0.05	0.34	0.895						
4. Drug-Biased Behavior Model				54.5	0.23	0.172	3 vs. 4	0.091	**0.035**
Verbal IQ	-0.46	0.04	0.264						
Regular opioid use (years)	-0.57	0.07	0.256						
Picture cue-induced craving	0.11	0.35	0.772						
Drug>pleasant explicit choice	0.81	0.00	**0.047**						

Table S11. Hierarchical logistic regression analysis for inpatient treatment dropout. For each logistic regression, the standardized coefficient estimates (β), standard errors (SE), and p-values are displayed for each predictor variable. Summary statistics (AIC, pseudo-R^2, and p-values) are displayed for each model vs. the null model. Increases in R^2 values and p-values are displayed for each step's model comparison. P-values below 0.05 are denoted in bold typeface.

References:
1. Sheehan DV, Lecrubier Y, Sheehan KH, Amorim P, Janavs J, Weiller E, et al. (1998): The Mini-International Neuropsychiatric Interview (M.I.N.I.): the development and validation of a structured diagnostic psychiatric interview for DSM-IV and ICD-10. The Journal of clinical psychiatry 59 Suppl 20: 22-33;quiz 34-57.
2. McLellan AT, Kushner H, Metzger D, Peters R, Smith I, Grissom G, et al. (1992): The Fifth Edition of the Addiction Severity Index. Journal of substance abuse treatment 9: 199–213.
3. Heatherton TF, Kozlowski LT, Frecker RC, Fagerstrom K (1991): The Fagerström Test for Nicotine Dependence: a revision of the Fagerstrom Tolerance Questionnaire. British Journal of Addiction 86: 1119–1127.
4. Beck AT, Ward CH, Mendelson M, Mock J, Erbaugh J (1961): An Inventory for Measuring Depression. Archives of General Psychiatry 4: 561–571.
5. Beck AT, Epstein N, Brown G, Steer RA (1988): An inventory for measuring clinical anxiety: psychometric properties. J Consult Clin Psychol 56: 893–897.
6. Gossop M, Griffiths P, Powis B, Strang J (1992): Severity of dependence and route of administration of heroin, cocaine and amphetamines. British Journal of Addiction 87: 1527–1536.
7. Handelsman L, Cochrane KJ, Aronson MJ, Ness R, Rubinstein KJ, Kanof PD (1987): Two New Rating Scales for Opiate Withdrawal. The American Journal of Drug and Alcohol Abuse 13: 293–308.
8. Tiffany ST, Singleton E, Haertzen CA, Henningfield JE (1993): The development of a cocaine craving questionnaire. Drug and Alcohol Dependence 34: 19–28.
9. Vafaie N, Kober H (2022): Association of Drug Cues and Craving With Drug Use and Relapse. JAMA Psychiatry 79: 641–650.
10. Goldstein RZ, Woicik PA, Moeller SJ, Telang F, Jayne M, Wong C, et al. (2010): Liking and wanting of drug and non-drug rewards in active cocaine users: the STRAP-R questionnaire. J Psychopharmacol 24: 257–266.
11. Huang Y, Ceceli AO, Kronberg G, King S, Malaker P, Parvaz MA, et al. (2024): Association of Cortico-Striatal Engagement During Cue Reactivity, Reappraisal, and Savoring of Drug and Non-Drug Stimuli With Craving in Heroin Addiction. American Journal of Psychiatry 181: 153–165.
12. Kronberg G, Ceceli AO, Huang Y, Gaudreault P-O, King SG, McClain N, et al. (2024): Shared orbitofrontal dynamics to a drug-themed movie track craving and recovery in heroin addiction. Brain awae369.
13. Costello AB, Osborne J (2005): Best practices in exploratory factor analysis: four recommendations for getting the most from your analysis [no. 1]. Practical Assessment, Research, and Evaluation 10. https://doi.org/10.7275/jyj1-4868
